# Supplementary material for: Whole genome DNA and RNA sequencing of whole blood elucidates the genetic architecture of gene expression underlying a wide range of diseases
Source: Sci Rep. 2022 Nov 23;12:20167. doi: 10.1038/s41598-022-24611-w (PMC9686236; doi:10.1038/s41598-022-24611-w)
Supplement: Supplementary file 2 — Supplementary Information 2. [file 41598_2022_24611_MOESM2_ESM.docx]

Supplemental Materials

Whole Genome DNA and RNA Sequencing of Whole Blood Elucidates the Genetic Architecture of Gene Expression Underlying a Wide Range of Diseases

Chunyu Liu^1,2,#^, Roby Joehanes^3,#^, Jiantao Ma^4^, Yuxuan Wang^1^, Xianbang Sun^1^, Amena Keshawarz^3^, Meera Sooda^3^, Tianxiao Huan^3^, Shih-Jen Hwang^3^, Helena Bui^3^, Brandon Tejada^3^, Peter J. Munson^3^, Cumhur Y. Demirkale^5^, Nancy L. Heard-Costa^2,6^, Achilleas N Pitsillides^1^, Gina M. Peloso^1^, Michael Feolo^7^, Nataliya Sharopova^7^, Ramachandran S. Vasan^2,6^, Daniel Levy^2,3,#^

^1^Department of Biostatistics, School of Public Health, Boston University, Boston, MA, USA

^2^Framingham Heart Study, Framingham, MA, USA

^3^Population Sciences Branch, Division of Intramural Research, National Heart, Lung, and Blood Institute, National Institutes of Health, Bethesda, MD, USA

^4^Nutrition Epidemiology and Data Science, Friedman School of Nutrition Science and Policy, Tufts University, Boston, MA, USA

^5^Critical Care Medicine Department, Clinical Center, National Institutes of Health, Bethesda, MD, USA

^6^Departments of Medicine and Epidemiology, Boston University Schools of Medicine and Public Health, Boston, MA, USA

^7^National Center for Biotechnology Information, Bethesda, MD

**Supplemental Table 1**. Participant characteristic

| **Variable**  **mean (SD) or %** | **Offspring cohort**  **(n=720)** | **Third Generation cohort**  **(n=1902)** |
| --- | --- | --- |
|  |  |  |
| Women | 58.6 | 52.3 |
| Age, years | 71.3 (8.2) | 46.5 (8.7) |
| BMI, kg/m2 | 28.5 (5.6) | 27.7 (5.6) |
| SBP, mmHg | 126.8 (16.7) | 115.9 (14.1) |
| DBP, mmHg | 73.6 (9.8) | 74.2 (9.4) |
| Fasting glucose, mg/dL | 105.7 (19.8) | 96.5 (19.7) |
| TC, mg/dL | 189.2 (36.4) | 186.3 (33.2) |
| HLD, mg/dL | 57.8 (18.7) | 60.0 (17.8) |
| Trig, mg/dL | 119.1 (73.5) | 110.4 (70.9) |
| LDL, mg/dL | 108.0 (31.9) | 104.2 (29.8) |
| Current smoking | 8.2 | 10.8 |
| Hypertension | 48.1 | 33.6 |
| Diabetes | 12.6 | 5.7 |
| HRX | 44.1 | 19.9 |
| LIPIDRX | 41.4 | 29.0 |
| DMRX | 9.1 | 5.2 |

BMI, body mass index; SBP/DBP, systolic/diastolic blood pressure; TC, total cholesterol; HDL, high density lipoprotein; Trig, triglyceride; LDL, low-density lipoprotein; HRX, treatment for hypertension; LIPIDRX, treatment for high lipid level; DMRX, treatment for diabetes.

**Supplemental Table 2**. *Cis*- and *trans*-eQTL in the Framingham Heart Study

|  | eQTLs at gene level | lncRNA eQTLs |
| --- | --- | --- |
| Cis-eQTL-eGene (*p* < 5e-8) | | |
| Number of pairs | 6,778,286 pairs | 442,379 *cis*-eQTLs are located in 1518 *cis*-lncRNAs genes |
|  | 2,855,111 unique cis-eQTLs and 15,982 eGenes |  |
| Trans-eQTL-eGene (*p* < 1e-12) | | |
| Number of pairs | 1,469,754 pairs | 117,862 *trans*-eQTLs are located in 475 *trans*-lncRNAs genes |
|  | 526,056 unique trans-eQTLs and 7,233 trans-eGenes |  |

**Supplemental Table 3.** Top cis-eQTLs (one eQTL variant per eGene)

**Supplemental Table 4.** Top trans-eQTLs (one eQTL variant per eGene)

**Supplemental Table 5.** Top cis-eQTLs in long nocoding RNAs (one eQTL variant per eGene)

**Supplemental Table 6.** Top trans-eQTLs in long nocoding RNAs (one eQTL variant per eGene)

**Supplemental Table 7.** Go analyses for eQTLs with p < 1e-7

**Supplemental Table 8.** GWAS enrichment analyses for cis-eQTLs

**Supplemental Table 9.** GWAS enrichment analyses for trans-eQTLs

**Supplemental Table 10.** Significant genes in MR analyses

**Supplemental Table 11.** MR analysis for COVID-19 GWAS genes

**Supplemental Table 12.** Replication of Battle's lead cis-eQTLs

**Supplemental Table 13.** Replication of Battle's lead trans-eQTLs


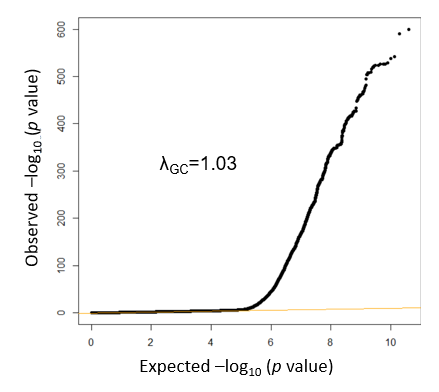


**Supplemental Figure 1**. Q-Q plot: –log *p* values of variant-gene pairs based on association analyses on chromosome 12.


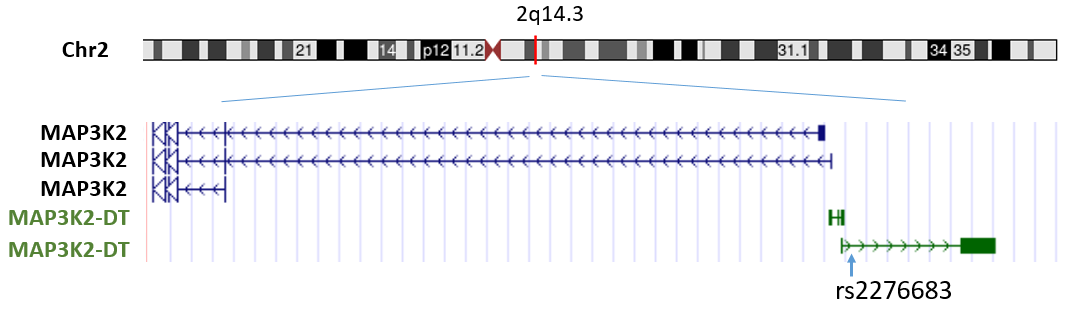


**Supplemental Figure 2**. Cis-long noncoding RNA, MAP3K2-DT, and the lead cis-eQTL, rs2276683.


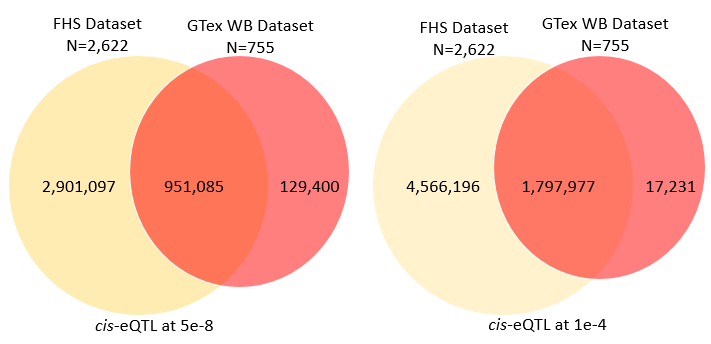


**Supplemental Figure 3**. Replication analyses using GTEx. The figure on the left was restricted to cis-eQTLs identified at 5e-8. The figure on the right was restricted to cis-eQTLs identified at 5e-4.


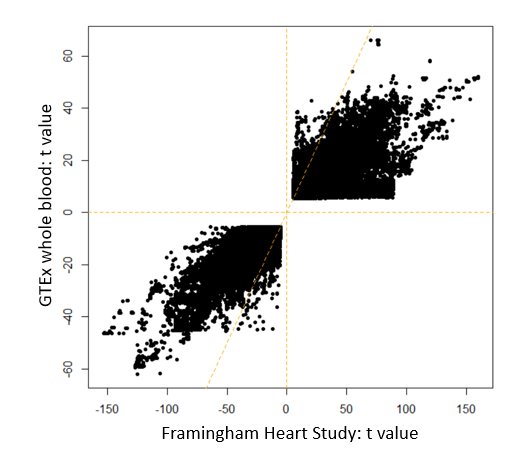


**Supplemental Figure 4**. Comparison of t-values of associations of cis-eQTL-eGene pairs identified in the Framingham Heart Study and the GTEx whole blood samples.
